# Small-scale field evaluation of transfluthrin-treated eave ribbons and sandals for the control of malaria vectors in rural Tanzania

**DOI:** 10.1186/s12936-023-04476-8

**Published:** 2023-02-04

**Authors:** Arnold S. Mmbando, Winifrida P. Mponzi, Halfan S. Ngowo, Khamis Kifungo, Robert Kasubiri, Rukiyah M. Njalambaha, Tegemeo Gavana, Alvaro E. Eiras, Elis P. A. Batista, Marceline F. Finda, Onyango P. Sangoro, Fredros O. Okumu

**Affiliations:** 1 Environmental Health and Ecological Sciences, Ifakara Health Institute, Ifakara, Tanzania; 2 School of Public Health, Faculty of Health Sciences, University of the Witwatersrand, Parktown, Republic of South Africa; 3 Institute of Biodiversity, Animal Health and Comparative Medicine, University of Glasgow, Glasgow, UK; 4 Laboratory of Technological Innovation of Vector Control, Department of Parasitology, Biological Science Institute, Federal University of Minas Gerais, Belo Horizonte, Brazil; 5 Human Health Theme, International Centre of Insect Physiology and Ecology (ICIPE), Nairobi City, Kenya; 6 School of Life Science and Bioengineering, Nelson Mandela African Institution of Science & Technology, Arusha, Tanzania

## Abstract

**Background:**

Early-evening and outdoor-biting mosquitoes may compromise the effectiveness of frontline malaria interventions, notably insecticide-treated nets (ITNs). This study aimed to evaluate the efficacy of low-cost insecticide-treated eave ribbons and sandals as supplementary interventions against indoor-biting and outdoor-biting mosquitoes in south-eastern Tanzania, where ITNs are already widely used.

**Methods:**

This study was conducted in three villages, with 72 households participating (24 households per village). The households were divided into four study arms and assigned: transfluthrin-treated sandals (TS), transfluthrin-treated eave ribbons (TER), a combination of TER and TS, or experimental controls. Each arm had 18 households, and all households received new ITNs. Mosquitoes were collected using double net traps (to assess outdoor biting), CDC light traps (to assess indoor biting), and Prokopack aspirators (to assess indoor resting). Protection provided by the interventions was evaluated by comparing mosquito densities between the treatment and control arms. Additional tests were done in experimental huts to assess the mortality of wild mosquitoes exposed to the treatments or controls.

**Results:**

TERs reduced indoor-biting, indoor-resting and outdoor-biting *Anopheles arabiensis* by 60%, 73% and 41%, respectively, while TS reduced the densities by 18%, 40% and 42%, respectively. When used together, TER & TS reduced indoor-biting, indoor-resting and outdoor-biting *An. arabiensis* by 53%, 67% and 57%, respectively. Protection against *Anopheles funestus* ranged from 42 to 69% with TER and from 57 to 74% with TER & TS combined. Mortality of field-collected mosquitoes exposed to TER, TS or both interventions was 56–78% for *An. arabiensis* and 47–74% for *An. funestus*.

**Conclusion:**

Transfluthrin-treated eave ribbons and sandals or their combination can offer significant household-level protection against malaria vectors. Their efficacy is magnified by the transfluthrin-induced mortality, which was observed despite the prevailing pyrethroid resistance in the study area. These results suggest that TER and TS could be useful supplementary tools against residual malaria transmission in areas where ITN coverage is high but additional protection is needed against early-evening and outdoor-biting mosquitoes. Further research is needed to validate the performance of these tools in different settings, and assess their long-term effectiveness and feasibility for malaria control.

## Background

Current malaria interventions have contributed to significant gains in the past two decades, averting nearly 2 billion cases and 11 million deaths since 2000 [1]. However, the efforts are evidently reaching their limits, and malaria cases have recently been on the rise in many high-burdened countries [1]. Vector control tools, such as insecticide-treated nets (ITNs) and indoor residual spraying (IRS), which together contributed to an estimated 79% of malaria reduction between 2000 and 2015 [2], are becoming limited by factors such as outdoor-biting, resistance to commonly used insecticides [3] and reduced ITN durability under field conditions [4, 5].

These challenges are further compounded by poor housing conditions in rural and semi-urban communities where malaria burden is highest [6, 7], as well as human activity and behaviours that drive transmission [8]. Malaria vectors prefer to enter houses via the eave-spaces [9] and most malaria transmission in Africa is associated with mosquitoes that bite indoors [10].

Measures targeted at eave spaces can, therefore, protect individuals when indoors but not under bed nets, thereby complementing current interventions and reducing the malaria control gaps [7]. Several studies have shown the potential of housing improvement for reducing malaria risk [7, 11, 12]. However, this concept still faces concerns among key stakeholders, especially with regard to the perceived high costs as well as difficulties of implementing such a strategy [13, 14].

Spatial repellents are one of the complementary vector control tools, that offer protection against mosquitoes and mosquito-borne diseases. They are generally in the form of volatile chemicals, which can be actively or passively dispensed into the air to repel or kill host seeking mosquitoes. Low-cost, easy-to-use and easy-to-scale spatial repellent products have been proposed and demonstrated as potential candidates to address protection gaps associated with mosquitoes that bite outdoors, early in the evenings or after bed times [15–17].

Studies in east Africa have previously shown that eave ribbons made of hessian materials and treated with transfluthrin (a vapour-phase pyrethroid that is both a spatial repellent and toxicant) can provide significant protection against both indoor-biting and outdoor-biting mosquitoes [15, 18, 19]. This technology has the added advantage over conventional house screening in that it can be deployed even on poorly-constructed house structures, including even the makeshift houses commonly used by itinerant farmers in east Africa, without the need to first modify the structures [18]. Another related technology, transfluthrin-treated sandals, has also been developed and assessed against both day-biting and night-biting mosquitoes [20]. In semi-field experiments in Tanzania, transfluthrin-treated sandals offered 72% protection against the malaria vector, *Anopheles arabiensis* and 35% protection against the dengue vector, *Aedes aegypti* [20].

In Brazil, the same sandals offered more than 75% protection against *Anopheles darlingi* and more than 50% reduction against *Ae. aegypti* bites (Eiras A., et al., pers.commun.) It is anticipated that transfluthrin-treated sandals could ensure round-the clock protection when deployed in areas where people already have ITNs. However, neither the transfluthrin-treated eave ribbons nor the sandals have been evaluated in real world settings to validate their protective efficacies demonstrated in the controlled environments.

This study, therefore, aimed to assess the effectiveness of these two interventions, either singly or in combination, against indoor- and outdoor-biting mosquitoes in malaria-endemic villages in south-eastern Tanzania, where ITNs are already widely used and malaria vectors are resistant to pyrethroids.

## Methods

### Study area

**Fig. 1:**
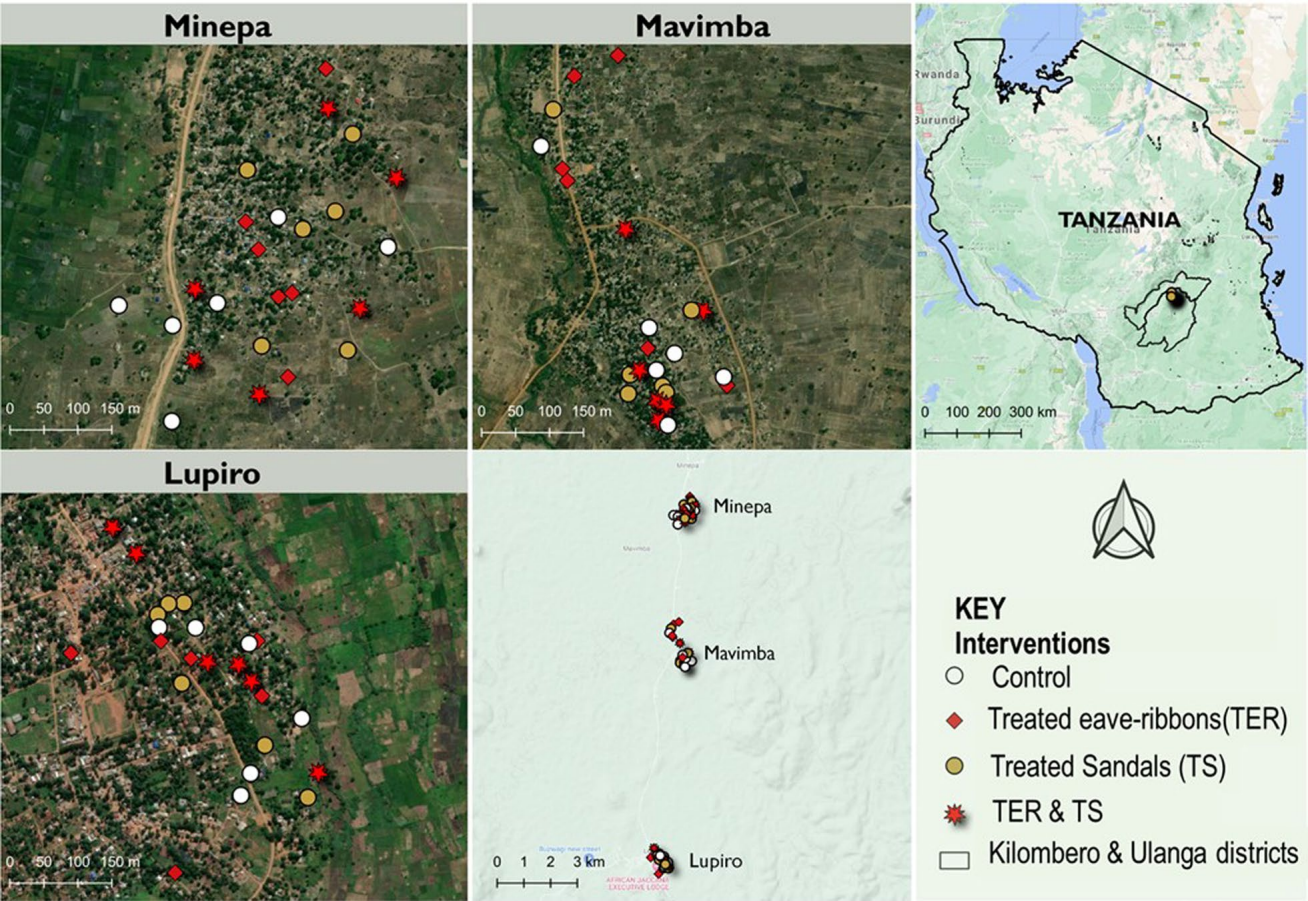
Map of the study villages in Tanzania

This study was conducted in three rural villages in Ulanga district in Tanzania (Minepa; 8.2710^o^S and 36.6771^o^E, Lupiro; 8.385^o^S and 36.670^o^E and Mavimba; 8.3124^o^S and 36.6771^o^E villages) (Fig. 1), between February to September 2018. Residents here are crop farmers, cultivating crops mainly rice and maize farmers [21]. Annual rainfall is 1200–1600 mm and mean daily temperatures were 20.0–32.6 °C [18]. The primary malaria vectors in the area are *An. arabiensis* and *Anopheles funestus*, the latter mediating most of the transmission [22, 23]. ITNs are the primary malaria control tools, and are mostly distributed through government agencies [24]. Local populations of the primary malaria vectors are known to be resistant to insecticides used for malaria control, especially pyrethroids and carbamates [23, 25].

### Study procedures

#### Preparation of transfluthrin-treated products

Preparation of the transfluthrin-treated eave ribbons and transfluthrin-treated sandals was done at the Vector Control Product Fabrication Facility (*Ifakara Mozzie House*) at Ifakara Health Institute. Treatments were done in a specially-designed containment unit, so that all waste could be appropriately disposed of.

#### Transfluthrin-treated eave-ribbons

**Fig. 2:**
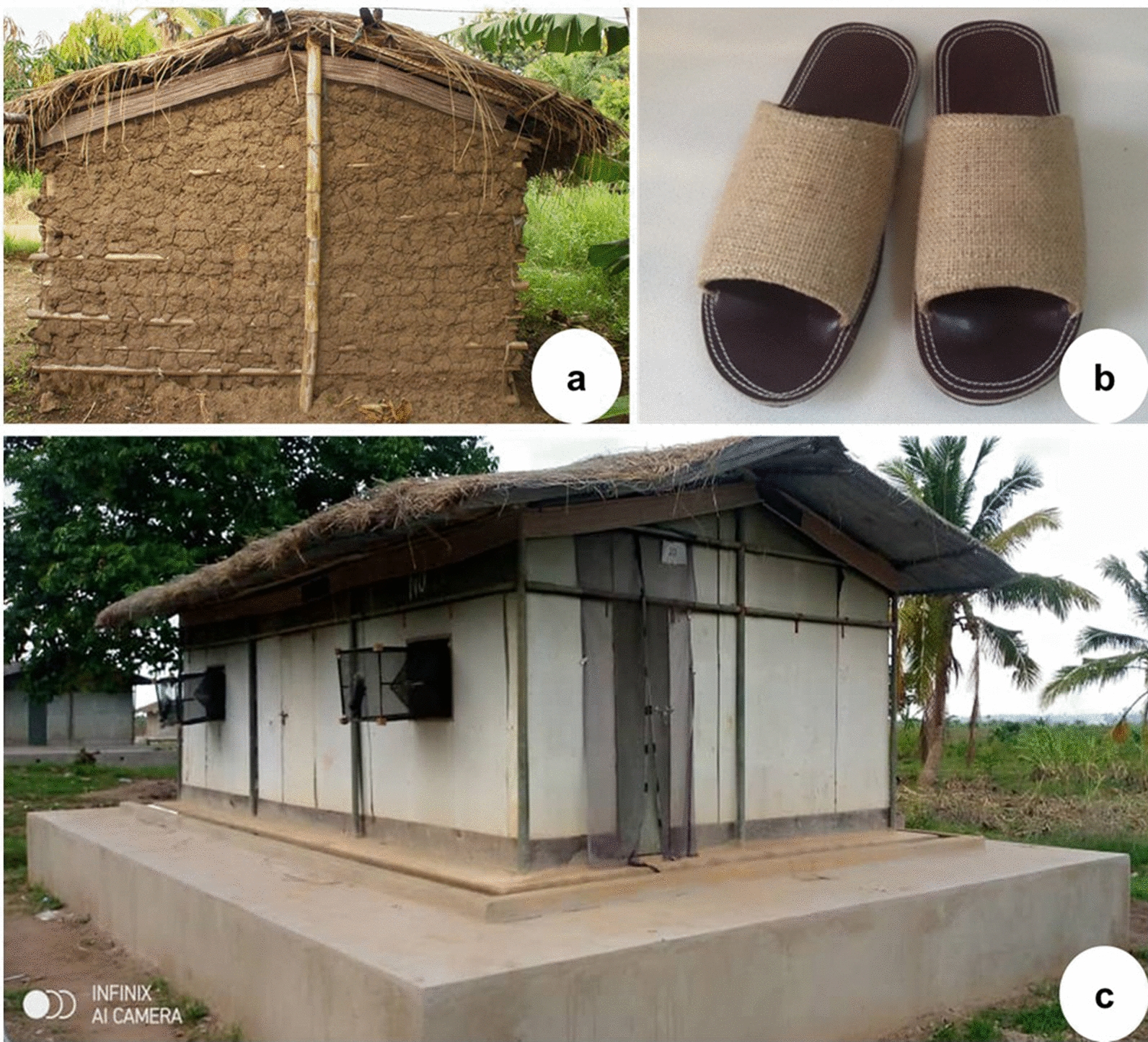
Pictures of houses fitted with transfluthrin-treated eave ribbons: **a** Poorly-finished mud-walled and thatch-roofed house with large eave gaps and **b** Brick-walled metal-roofed house. **c** The transfluthrin-treated sandals, and **d** Ifakara experimental huts used in the assessment of mosquito mortality effects

The eave ribbons were designed as rectangular pieces of hessian (0.15 m wide × 25 m long), treated with a transfluthrin formulation and wrapped around eave spaces of houses, without closing the eave gaps as previously described [17, 18]. The eave ribbons technology exploits the house-entry behaviour of mosquitoes by delivering a vapour-phase pyrethroid that has both a repellence and toxic effect thus protecting users both indoors and outdoors from mosquito bites [18, 19]. The ribbons were treated with a 1.5% solution achieving a concentration of 4 g/m^2^ of transfluthrin as described in previous studies [17, 18]. After treatment, the ribbons were dried under a shade prior to deployment. The houses randomized to the respective study arm were fitted with the treated ribbons around the eave-spaces as previously described [17, 18] and the ribbons left in situ for the duration of the study (Fig. 2a).

#### Transfluthrin-treated sandals

The sandals were designed and manufactured as previously described by Sangoro et al. [20]. Each pair was affixed with a hessian fabric measuring 395cm^2^ and treated with 8% transfluthrin solution, yielding a concentration of 20.3 g/m^2^ of transfluthrin. Each household randomized to treated sandal arm received two pairs of treated sandals for every three adults. In line with the risk assessment done on the test products [26], no sandal was given to any children below two years old. The users were advised to wear the sandals outdoors and indoors and place them near their sleeping spaces whenever they went to bed to sleep (Fig. 2b). Details of the sandals and their application are described elsewhere [20].

#### Household selection and mosquito trapping

In each of the three study villages, 24 houses (totaling 72 houses) were selected from a list of households provided by the community leaders and recruited upon written informed consent. Only houses with open eave-spaces were recruited into the study, otherwise the next house in the random list was considered. The minimum distance between the individual sampled houses was 50 m to enhance independence of the houses and minimize likelihood of mosquitoes being diverted between the treatment and control houses. The selected households were then randomly divided into four groups of six houses each village and assigned to different study arms, which received either transfluthrin-treated eave ribbons (TER), transfluthrin-treated sandals (TS), a combination of TER and TS and control which no treatment was assigned. This way, each intervention arm had a total of 18 houses (Table 1). Basic training on the use and handling of the sandals and ribbons was provided to all users in the houses. All study houses were provided with a pair of ITNs (Olyset™, Sumitomo Chemical, Arusha, Tanzania), to ensure full coverage with current primary vector control tool in the study area.

**Table 1:** Distribution of households in the four intervention arms

Group	Intervention	^a^Trap nights	^a^Households per village	^a^Households in three study villages
1	TS only	72	6	18
2	TER only	72	6	18
3	TER & TS	72	6	18
4	LLINs (control)	72	6	18

^a^Each intervention was tested for three rounds in comparison to the controls in each village. Thus there were 216 trapping nights per intervention arm

Once the interventions were assigned, mosquito sampling was done for a total of 216 trap-nights per study arm. Considering the nightly catches of 13 *An. arabiensis* mosquitoes per night per house, observed in pilot trials, this sample size of 216 trap nights per study arm was determined as sufficient to achieve an 80% statistical power, to detect reductions in mosquito biting densities observed in previous experimental hut evaluations of eave ribbons [17, 27].

#### Mosquito sampling

Mosquito collections were done using three methods. Outdoor-biting rates were estimated using the miniaturized double net trap (DN-Mini trap) from 1900 to 2200 h (with a consenting, adult male volunteer inside the DN-Mini trap) [28], to mimic natural outdoor activity in the communities. Indoor host-seeking mosquitoes were sampled using the CDC light trap [29] from 2200 to 0630 h and lastly, indoor resting mosquitoes were sampled using the Prokopack^®^ aspirators [30] the next morning between 06:40 to 07:30 h. The sampling schedule consisted of deploying all the three traps in the same house as outlined above. Sampling was done in eight households per night, and repeated for three nights each month in each village to complete one round of collection covering all the 24 houses in each village; reaching 72 monthly collections per village. Three rounds of sampling were completed in each village such that at the end of the survey, each house had been sampled nine times by CDC light traps, nine times by DN-Mini traps and nine times by Prokopack^®^ aspirators (Table 1).

#### Laboratory analysis of collected mosquitoes

*Anopheles* mosquitoes collected during the study were killed and sorted by taxa and physiological status. The blood-fed mosquitoes were separated and analysed individually by enzyme-linked immunosorbent assays (ELISA) to determine their blood meal sources [31]. The unfed ones were pooled in tens for detection of *Plasmodium* sporozoite infections. Sub-samples of primary malaria vectors was subjected to species identification using multiplex PCR to distinguish between members of *Anopheles gambiae* complex and *An. funestus* group [32, 33].

#### Assessing mosquito mortality associated with the transfluthrin-treated eave ribbons and sandals in experimental huts

A supplementary study was done in eight experimental huts (Ifakara design [34]) located in Lupiro village to evaluate the bio-efficacy of TERs and TS for killing mosquitoes. The design and applications of these experimental huts have been described in previous studies [17, 34]. Huts used for assessments of TS, TER and controls were situated at least 50 m apart to minimize interaction of effects of treatments in different huts. Each intervention (TER only, TS only, TER & TS, and control) were assigned to two experimental huts each night. In the treatment arm with TERs, the ribbons were fitted around the eave-gaps without closing the eave spaces.

The mosquitoes were then collected by a volunteer sitting inside the DN-Mini trap placed 5 m from the huts until 2200 h. Similarly, in the treatment arm with TS, the volunteers wore the treated sandals and sat inside the DN-Mini trap, outdoors in the peri-domestic space of the house until 2200 h, after which they went indoors and kept the sandals inside the huts beside the bed for the rest of the night. In the treatment arm with TER & TS, the TER was wrapped along the eave-gaps of the huts and the male volunteers wore the TS and sat inside the DN-Mini traps outdoor until 2200 h, then they entered the huts and placed the TS indoors beside the bed for the rest of the night. Two adult male volunteers slept under an LLIN (Olyset™) inside each hut including the controls. The two control huts were left without TER or TS, but had a LLIN, which was standard in all the huts.

In this experiment, mosquito collections were done using window-exit traps to catch indoor mosquitoes attempting to exit the huts [17], DN-Mini traps for outdoor-biting mosquitoes from 1900 to 2200 h [28] and Prokopack^®^ aspirators for resting mosquitoes each morning [30]. The exit traps offered a passive mechanism to collect mosquitoes and retain them temporarily for further observations without excessive mortality due to handling, such as often seen with CDC light traps. Four nights of testing was done each week, for a total of four weeks. The participating volunteers sat inside the DN-Mini traps from 1900 to 2200 h at the peri-domestic area of the huts, and afterwards went inside the huts to sleep under the nets. After every four nights of testing the interventions were rotated to different huts, ensuring three days in between to minimize residual effects of the treatments [17]. The collected mosquitoes were kept in a nearby insectary in the same village; and maintained on 10% glucose solution for 24 h monitoring (Fig. 2c).

### Data analysis

Data were analysed and processed using open-source statistical software, R version 3.5.0 [35]. Mosquito count data collected both indoors and outdoors in the treatment and control houses were modelled using Generalized Linear Mixed model (GLMM) following a negative binomial distribution [36]. Negative binomial distribution was used to account for the over-dispersion which cannot be corrected with the Poisson distribution [37]. In this model, the response variable was the number of mosquitoes captured while the main fixed variable was the interventions used. Random terms were included to account for the pseudo replication and unexplained variation between villages, volunteer ID and day of collection. Each species and methods of collection were analysed separately. The means and sums were used to assess the sporozoites rates, blood feeding index and 24 h mortality between control and the intervention arms.

## Results

Overall, both transfluthrin-treated eave ribbons and transfluthrin-treated sandals, when used as complementary interventions alongside ITNs, were effective at reducing mosquito biting and resting densities compared to houses that had only ITNs. The following section provides details of their performance against outdoor-biting, indoor biting and indoor resting mosquitoes.

### Outdoor-biting mosquito densities

**Fig. 3:**
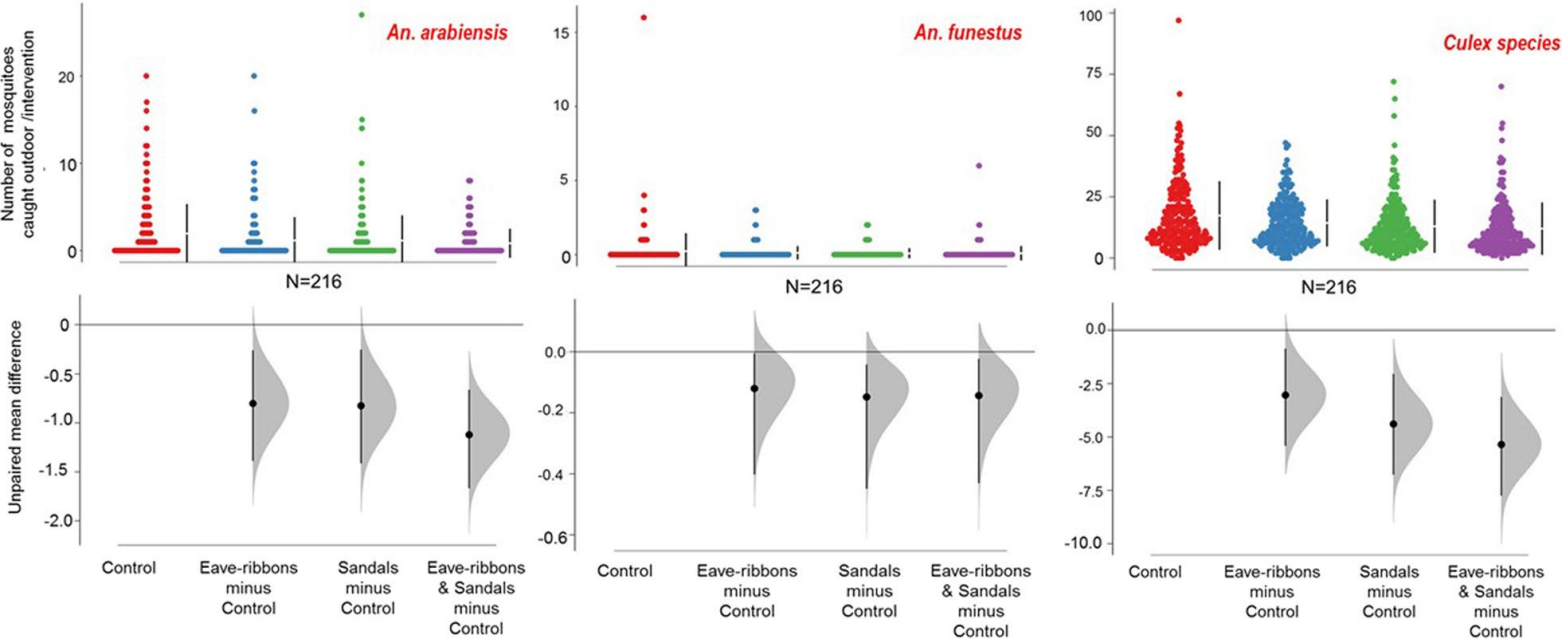
Reduction in outdoor-biting by *An. arabiensis*, *An. funestus* and *Culex species,* as sampled using the miniaturized double net traps (DN-Mini) in the treatment and control houses

The effects of the interventions on outdoor-biting are shown in Tables 2 and 3 and in Fig. 3. When TERs were tested alone, there was a 41% reduction in outdoor-biting against *An. arabiensis,* a 50% outdoor biting reduction against *An. funestus*, 17% outdoor biting reduction against *Culex* mosquitoes and 67% outdoor biting reduction against *Mansonia* species relative to the controls. On the other hand, TS alone resulted in an outdoor-biting reduction of 42%, 59% and 25% by *An. arabiensis, An. funestus* and *Culex* spp., respectively. When both TER and TS were combined in the same house, outdoor-biting was reduced by 57%, 59%, 32% and 67% against *An. arabiensis*, *An. funestus*, *Culex* spp. and *Mansonia* spp., respectively.

**Table 2:** Effects of transfluthrin-treated products on reduction of indoor and outdoor densities of malaria vectors

Intervention	Mosquito taxa	Host seeking mosquitoes [indoors]			Resting mosquitoes [indoors]			Host-seeking mosquitoes [outdoors]		
		CDC-light traps			Prokopack^®^ aspirators			Double net mini (DN-mini)-trap		
		Total no. mosquitoes caught	RR [LRR-URR]	Percentage (%) reduction	Total no. mosquitoes caught	RR [LRR-URR]	Percentage (%) reduction	Total no. mosquitoes caught	RR [LRR-URR]	Percentage (%) reduction
Control	*An. arabiensis*	2237	Ref.	–	142	Ref.	–	424	Ref.	–
Transfluthrin-treated eave ribbons (TER)		798	0.40**	60	35	0.27**	73	251	0.59**	41
			[0.29, 0.56]			[0.26, 0.28]			[0.40, 0.88]	
Transfluthrin-treated sandals (TS)		1785	0.82	18	78	0.60**	40	246	0.58**	42
			[0.58, 1.15]			[0.59, 0.61]			[0.39, 0.87]	
Transfluthrin-treated eave-ribbons & sandals (TER & TS)		1069	0.47**	53	38	0.33**	67	182	0.43**	57
			[0.34, 0.65]			[0.32, 0.33]			[0.29, 0.65]	
Control	*An. funestus*	582	Ref.	–	17	Ref.	–	51	Ref.	–
Transfluthrin-treated eave ribbons (TER)		452	0.57**	42	5	0.31	69	25	0.50**	50
			[0.37, 0.87]			[0, 0.09]			[0.24, 1.05]	
Transfluthrin-treated sandals (TS)		422	0.73	27	4	0.26**	74	19	0.41**	59
			[0.47, 1.1]			[0.07, 0.92]			[0.19, 0.88]	
Transfluthrin-treated eave-ribbons & sandals (TER & TS)		434	0.43**	57	4	0.26**	74	20	0.41**	59
			[0.46,1.13]			[0.07, 0.84]			[0.19, 0.87]	

^**^ = P < 0.05, RR [LRR, URR] = Relative risks with its 95% confidence intervals, Study was conducted for a total of 216 trapping nights

**Table 3:** Effects of transfluthrin-treated products on reduction of indoor and outdoor densities of other mosquito species

Intervention	Mosquito taxa	Host seeking mosquitoes [indoors]			Resting mosquitoes [indoors]			Host-seeking mosquitoes [outdoors		
		CDC-light traps			Prokopack^®^ aspirators			Double net mini (DN-mini)-trap		
		Total no. mosquitoes caught	RR [LRR-URR]	Percentage (%) reduction	Total no. mosquitoes caught	RR [LRR-URR]	Percentage (%) reduction	Total no. mosquitoes caught	RR [LRR-URR]	Percentage (%) reduction
Control	*Culex spp*	9316	Ref.	–	2925	Ref.	–	3748	Ref.	–
Transfluthrin-treated eave ribbons (TER)		6865	0.78**	22	2140	0.83	17	3092	0.83**	17
			[0.65, 0.94]			[0.67, 1.03]			[0.72, 0.95]	
Transfluthrin-treated sandals (TS)		9996	1.07	− 7	2392	0.93	7	2799	0.75**	25
			[0.88, 1.29]			[0.75, 1.15]			[0.65, 0.86]	
Transfluthrin-treated eave-ribbons & sandals (TER & TS)		6549	0.73**	27	2269	0.82	18	2592	0.68**	32
			[0.60, 0.88]			[0.67, 1.02]			[0.60, 0.80]	
Control	*Mansonia* spp	76	Ref.	–	10	Ref.	–	21	Ref.	–
Transfluthrin-treated eave ribbons (TER)		7	0.10**	90	4	0.23	79	7	0.33**	67
			[0.03, 0.34]			[0.03, 1.67]			[0.09, 1.26]	
Transfluthrin-treated sandals (TS)		61	0.91	9	2	0.16	85	42	2	− 100
			[0.37, 2.21]			[0.02, 1.32]			[0.63, 6.31]	
Transfluthrin-treated eave-ribbons & sandals (TER & TS)		24	0.40	60	5	0.16	86	7	0.33	67
			[0.15, 1.05]			[0.01, 1.77]			[0.09, 1.26]	

** represents statistical significant difference of mosquito biting reduction observed between the interventions and the control. i.e. p<0.05

### Indoor-biting mosquito densities

**Fig. 4:**
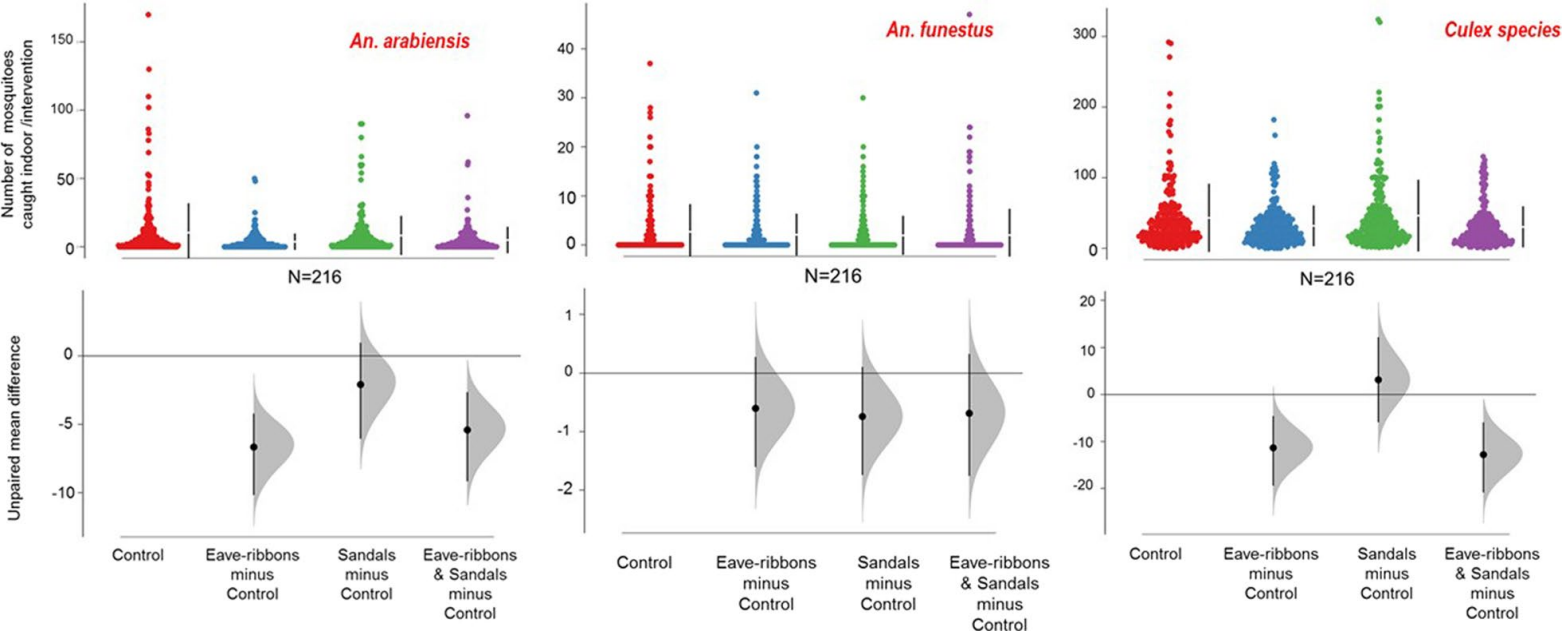
Reduction in indoor-biting *An. arabiensis*, *An. funestus* and *Culex* mosquitoes, as sampled using the CDC light traps in treated and control houses

Compared to the control houses, households with TER alone had 60% reduced indoor-biting by *An. arabiensis*, 90% reduce biting by *Mansonia* species and 42% against *An. funestus*. However, there was only a marginal reduction of 22% in indoor biting by *Culex* spp. Using TS alone also had yielded only marginal reductions in indoor biting by all mosquito species caught (Table 3). However, when TER and TS were used together in the same households, there was a 53% reduction of indoor-biting by *An. arabiensis*, 27% reduction of *Culex* species indoor bites, a 60% reduction of indoor-biting by *Mansonia* mosquitoes and a 57% reduction of biting by *An. funestus* (Table 2 and 3, Fig. 4).

### Indoor-resting mosquito densities

Presence of TER alone reduced indoor-resting *An. arabiensis* by 73%, *An. funestus* by 69%*, Culex* spp. by 17% and *Mansonia* spp. by 79%. TS alone also reduced the indoor-resting densities of *An. arabiensis* by 40%, *An. funestus* 74%, *Culex* spp 7% and *Mansonia* spp 85%. Lastly, when the two were combined, the indoor-resting densities of *An. arabiensis* reduced by 67%, *An. funestus* by 74%, *Mansonia* spp 86% and *Culex* spp 18% (Tables 2 and 3).

### Mosquito mortality

Mortality rate of *An. arabiensis* in the control huts where only LLINs were used was 11% within 24 h, whereas the mortality rate increased to 56% with TS, 74% with TER and 78% with both TER & TS. The huts with TER alone demonstrated a higher mortality rate of 76% against *An. funestus* compared to 16% in the control huts. Increased mortality was also observed in *Culex* species, from 15% in control huts to 70% when TER & TS was tested in a single hut (Table 4).

**Table 4:** Effects of transfluthrin-treated products on the densities of mosquitoes entering experimental huts with these products (estimated as repellency) and the percentage surviving the exposures (estimated as 24 h mortality)

Taxa	Intervention/control arm	No. mosquitoes caught	Percentage (%) reduction (repellency)	Total No. dead after 24 h	Percentage (%) dead after 24 h
*Anopheles arabiensis*	Control	629	Ref.	72	11
	Transfluthrin-treated sandals (TS)	380	40	213	56
	Transfluthrin-treated eave-ribbons (TER)	27	97	20	74
	Transfluthrin-treated eave-ribbons & sandals (TER &TS)	49	92	38	78
*Anopheles funestus*	Control	60	Ref.	10	16
	Treated sandals	36	40	17	47
	Treated eave-ribbons	75	− 25	57	76
	Treated eave-ribbons & sandals (TER & TS)	64	− 6	45	70
*Culex* spp.	Control	636	Ref.	94	15
	Treated sandals	455	28	266	58
	Treated eave-ribbons	197	69	123	63
	Treated eave-ribbons & sandals (TER & TS)	405	36	248	61

### Species identification

Overall PCR amplification rate was 85.8% (254/296). A majority of the amplified *An. funestus* mosquitoes were identified as *An. funestus *sensu stricto (*s.s.)* (97% (n = 246/254) and 3% (n = 8/254) were *Anopheles rivulorum*. On the other hand, all of the sub-samples of *An. gambiae *sensu lato (*s.l*.) were *An. arabiensis* (100% (n = 300/300).

### Blood meal sources

There were a total of 49 blood-fed mosquitoes collected indoors from both intervention and control households (44 *An. arabiensis* and 5 *An. funestus*). Analysis of the blood-meals by ELISA showed that 61% of the *An. arabiensis* (n = 27) had fed on humans, 34% on cattle (n = 15) and 5% on dogs (n = 2). All of the 5 blood-fed *An. funestus s.s.* mosquitoes had obtained their blood meals from cattle. Further analysis showed that 61% (n = 30) of blood-fed mosquitoes were caught in control houses. There was an 80% reduction of blood-fed mosquitoes in houses with TER and a 57% reduction in houses with TS alone. No blood-fed mosquitoes were caught in households with TER and TS together.

### Plasmodium infections

Only five *Anopheles* mosquitoes were found infected with *Plasmodium falciparum* sporozoites, thus it was not possible to compare effects of intervention on the infection rates. Four (4) of these were *An. funestus s.s.* and one (1) was *An. arabiensis*. All of these were captured indoors; two (2) from houses with TS, two (2) from control houses and one (1) from a house with TER.

## Discussion

As malaria burden has not decreased, or in some cases risen in recent years [38], there is need for new approaches or interventions to supplement the ongoing efforts. The currently available tools face multiple challenges of high cost, limited evidence of effectiveness or poor accessibility [13, 39]. This study aimed to evaluate the effectiveness of low-cost insecticide-treated eave ribbons and sandals as supplementary interventions against indoor-biting and outdoor-biting mosquitoes in rural Tanzanian villages where ITNs are already in use. Generally, the results showed that both interventions were effective at reducing mosquito biting and resting densities, and could potentially be useful in areas where ITN coverage is high but additional protection is needed against early-evening and outdoor-biting mosquitoes.

Transfluthrin-treated eave ribbons (TERs) reduced both indoor-biting and outdoor-biting densities of major malaria vectors when used in home that already had ITNs. In addition, there was significant mortality, exceeding 50% in most settings. This study therefore validates findings of previous semi-field and experimental hut trials, as well as the small-scale field assessments of the TERs alone in residential and make-shift farm houses in the same study area, which all of which generally showed greater than 70% protection [17, 18, 40]. The significant reduction of outdoor-biting by *An. arabiensis* and *An. funestus* mosquitoes confirm that the technologies may be used to address gaps in biting protection at times when human activity is substantial outdoors [41, 42], and presents a potential alternative for targeting residual malaria transmission.

The positive effects of treated eave ribbons on *An. funestus* densities (Table 2) were particularly interesting as this vector species has been particularly challenging in this area [43]. This species is highly endophilic and endophagic, and is known to mediate most of the persistent malaria transmission in the study area [22, 23]. Similar findings have also been observed in previous studies where *An. funestus* densities, despite being less responsive to transfluthrin-treated eave-ribbons than *An. arabiensis*, were still significantly impacted [17, 18]. Indeed, *An. funestus* in this area has far higher levels of pyrethroid resistance compared to *An. arabiensis* mosquitoes [25, 27], which could explain the slightly lower performance of transfluthrin-based tools against the species. The findings of this current study nonetheless highlights the TER to offer significant protection in areas dominated by *An. funestus* and *An. arabiensis*, despite pyrethroid resistance. Moreover, it shows the need for future eave ribbon designs to be treated with multiple active ingredients with different modes of action.

Despite having generally lower efficacy than the eave ribbons, the transfluthrin-treated sandals also provided substantial protection against *An. arabiensis* and *An. funestus* outdoors*.* This feature fits well with behaviours of residents in these malaria endemic settings, who tend to spend most of their evening hours outdoors, often without any protection against mosquito bites [44]. This approach also takes advantage of the proclivities of malaria vectors to bite at this time and to bite the lower extremities [20, 45, 46]. The treated sandals are therefore an ideal product for offering personal protection for users when other control tools cannot be used, e.g. in early evenings before bed time. The fact that the sandals can be used anywhere is an added advantage in ensuring compliance, as users do not have to be confined within a particular place. The sandals have also demonstrated for protection against daytime-biting mosquitoes such as *Aedes* species, a feature that is lacking in most other vector control tools. One previous study in a controlled environment demonstrated greater than 70% biting reduction against *An. arabiensis* and 50% biting reduction for *Ae. aegypti* outdoors [20]. In another study done in the semi-field settings with laboratory reared mosquitoes, transfluthrin-treated sandals reduced *An*. *arabiensis* by 54–86% and *Ae*. *aegypti* by 32–39%, without changing the overall distribution of bites on the body of the volunteers [45]. The apparent greater efficacy of the eave ribbons over the sandals is likely due to the greater surface area of the treated substrates; the average surface area for the eave ribbons was 3.75 m^2^ compared to 395 cm^2^ for the sandals. In addition, eaves represent the primary mosquito entry point into houses [47], which would allow greater interactions and contacts between mosquitoes and this technology compared to the sandals.

While the reduction in biting observed in this field study were moderate, compared to previous tests in controlled semi-field settings [17, 40], it was clear that the potential can be magnified by the transfluthrin-associated mortality against mosquitoes from the same study area (Table 4). The observed high mortality is particularly important given that mosquitoes in this area are known to be moderately to strongly resistant to the pyrethroids currently used for vector control [25, 48]. This suggests that evaluation of transfluthrin-based products should consider using insecticides with multiple modes of action, and also that there is likely to be additional communal protection affordable in addition to the personal and household level protection. Such mortality has also been observed in previous studies, which assessed the modes of action of spatial repellents on mosquitoes [49]. Here, it was suggested that the products induce feeding inhibition, repellency and also mortality on mosquitoes. The highest mosquito mortality in this current study were observed against *An. arabiensis* followed by *An. funestus;* while *Culex* and *Mansonia* mosquito species were only marginally impacted. The different mortality rates might be influenced by different level of resistance against pyrethroids as already demonstrated in the area [25, 27].

Combining both TER and TS in the same house offered modest increases in mosquito biting reductions for both indoor and outdoor as well as mortality in presence of the ITNs. The marginal increases of biting reductions seen was due to the increase of the repellence effects than when either TS or TER tested alone. Findings from this study showed that TS offer more of personal protections to individual when outdoors than indoors, while the TER shown to offer communal level protections to individuals at both indoors and outdoors. The additional value of combining both TER and TS at the same house was mainly seen outdoors when a volunteers wore the TS and stayed 5 m away from the house treated with TER. Thus, the slightly increase of the biting protections seen by combining the TER and TS at the same house was magnified by the TER which originally shown to offer significant biting reductions and mortality at both indoors and outdoors.

A recent modelling study demonstrates that an 10% increase of outdoor mosquito biting would result in an additional 10 million malaria cases [50]. This emphasizes the importance of developing tools that can be used to protect against outdoor mosquito bites. This study has demonstrated that TER in particular, and possibly TS can potentially address these gaps. It is therefore prudent that these tools are further developed and validated for use in low-income settings where malaria burden is unproportionate experienced. Beyond the possible applications against residual malaria transmission, these technologies may also be applicable in places with low but stalled malaria transmission, where additional interventions are needed to get to elimination; or places with especially high levels of outdoor biting and outdoor transmission. Indeed related technologies have already been tested for use in recreational settings [15, 27] and for creating mosquito-free outdoor spaces [27].

Added advantages of these technologies are their low-cost and ease of deployment, though centralized treatment of the units would be recommended to ensure environmental and health safeguards relevant to insecticide handling. Moreover, past evidence has indicated that transfluthrin-based hessian treatments, such as those used to manufacture the TERs and TS can remain effective for several months without re-treatment [15].

## Conclusion

This study evaluated the effectiveness of low-cost insecticide-treated eave ribbons and sandals as supplementary interventions against indoor-biting and outdoor-biting mosquitoes in rural Tanzanian villages where ITNs are already widely used; and where the dominant malaria vectors are resistant to key pyrethroid insecticides. The results showed that both interventions were effective at reducing mosquito biting and resting densities, and could potentially be useful in areas where ITN coverage is high but additional protection is needed against early-evening and outdoor-biting mosquitoes. Their efficacy is magnified by the transfluthrin-induced mortality despite the prevailing pyrethroid resistance. Overall, the efficacy of the ribbons was higher than the treated sandals. Efforts should be made to improve the designs of the eave ribbons and sandals, and future studies should evaluate their impact and feasibility for complementing current front-line malaria control interventions at larger scale.

## Data Availability

Data will be freely available from the lead author upon request.

## Abbreviations

TER: Transfluthrin-treated eave-ribbons
TS: Transfluthrin-treated sandals
CDC: Centre for disease control
CI: Confidence interval
GLMM: Generalized linear mixed effects model
IRS: Indoor residual spraying
IHI: Ifakara health institute
IRB: Institutional review board
ITNs: Insecticide-treated nets
NIMR: National Institute for Medical Research
PCR: Polymerase chain reaction
RR: Relative risks
DN-Mini trap: Miniaturized double-net trap
